# Dietary Assessment and Trends Among Preschoolers in South Korea: Data from KNHANES 2012–2021

**DOI:** 10.3390/nu18020240

**Published:** 2026-01-12

**Authors:** Yong-Seok Kwon, Ye-Jun Kim, Eun-Kyung Kim, Jin-Young Lee, Yangsuk Kim, Sohye Kim

**Affiliations:** 1 Department of Food Sciences, National Institute of Crop and Food Science, Rural Development Administration, Wanju-gun 55365, Republic of Korea; selenium2012@korea.kr (Y.-S.K.); backyul99@korea.kr (Y.-J.K.); ekkim20@korea.kr (E.-K.K.); cherry78@korea.kr (J.-Y.L.); kagatha@korea.kr (Y.K.); 2 Nutrition Care Services, Seoul National University of Bundang Hospital, Seongnam-si 13620, Republic of Korea; 3 Department of Medical Nutrition, Graduate School of East-West Medical Science, Kyung Hee University, Yongin-si 17104, Republic of Korea

**Keywords:** dietary assessment, dietary trend, preschooler, Korean, KNHANES

## Abstract

**Objective:** This study aims to investigate the dietary assessment and trends of preschoolers aged 3 to 5 years in Korea from 2012 to 2021 and to provide basic data for early childhood dietary education and policy development. **Methods:** Data from the Korea National Health and Nutrition Examination Survey (KNHANES) from 2012 to 2021 were analyzed for 2510 children in the 3–5 age group. Dietary intake was assessed using a 24 h recall. Intakes of food groups, dishes, and nutrients were calculated, and trends across years were tested using generalized linear models adjusted for gender, age, household income, energy intake, mother’s age, and mother’s education. **Results:** Over the tenyear period, intakes of carbohydrates, phosphorus, iron, sodium, potassium, carotene, thiamine, niacin, and vitamin C, as well as the carbohydrate energy ratio, showed significant declines. Meanwhile, protein, fat, retinol, and riboflavin increased, as did the protein and fat energy ratios. Fruit intake decreased by approximately 42 g among food group intakes. Analysis of foods contributing to total food intake revealed that milk, white rice, apples, and eggs consistently accounted for a high proportion of total intake in all survey years. Average calcium intake was approximately 100 mg below the estimated average requirement. **Conclusions:** The results of this study showed that preschoolers exhibit insufficient intake of certain nutrients, such as calcium, and a decrease in fruit intake. Interventions are needed to establish regular meal patterns, promote plant food intake such as fruit, and improve calcium intake. These results provide valuable evidence for designing dietary education programs and dietary guidelines tailored to early childhood.

## 1. Introduction

Early childhood is the period of the most rapid growth in the life cycle, and nutritional management of early childhood nutrition not only promotes normal growth and development but also forms an important foundation for an individual’s lifelong health, as it is a time of rapid physical growth and emotional and cognitive development [1,2,3,4,5,6]. Several studies have reported that nutrient intake during this period is associated with a higher prevalence of stunted growth, dental caries, and allergic diseases such as rhinitis, obesity, and anemia [7,8,9,10,11,12]. It has also been reported that dietary habits formed in early childhood are not easily changed and are strongly associated with health problems later in life [13,14,15,16]. Therefore, nutritional status in early childhood affects growth, cognitive development, and health throughout childhood, adolescence, and adulthood, and it is very important to establish good dietary habits and intake balanced nutrients [3,11,17].

Regarding studies on early childhood nutrition, Yu HC [4] reported that he conducted a basic study to improve early childhood nutrition by focusing on preschooler nutrition at home and mothers’ perceptions. Regarding food preferences, a study on eating behavior and food preferences according to the age and gender of infants and a study on the association between picky eating habits in infancy and growth were reported [3,16]. In addition, studies related to picky eating have shown that preschoolers are more likely to choose or prefer high-exposure foods and less likely to eat low-exposure foods [11,16,18,19].

Studies that have used national data to examine preschooler dietary habits include a study that examined the dietary status of Korean preschoolers using data from the KNHANES [11], and a study that examined the dietary behaviors, dietary supplements, and nutrient intakes of Korean preschoolers during feeding and weaning [17]. In addition, a study was reported that examined changes in the nutritional status of 3- to 5-year-olds attending childcare centers by comparing data from the 2010 and 2014 KNHANES [20]. In a study that investigated the zinc intake and nutritional status of preschoolers in Korea using KNHANES data from 2009 to 2013, 1.1% of all subjects consumed zinc below the estimated average requirement (EAR). Meanwhile, 10.7% of all subjects were reported to have consumed more than the UL (upper limit) intake [21].

Research on the dietary behaviors, food and nutrient intake, and dietary habits of preschool-age children in other countries reveals the following: A study using NHANES data from 2005 to 2012 analyzed food sources of total energy and 16 nutrients for 2740 infants and toddlers aged 0–24 months in the United States. Intakes of total fat, omega-3 fatty acids, and iron were lower than reference values, energy and protein intakes were higher than recommended, and a portion of the population exceeded the upper allowable intake limits for sodium, zinc, and retinol. Milk, 100% juice, and grain-based blends were reported as important sources of energy, especially during infancy [22]. Meanwhile, in Mexico, the 2012 Mexican National Health and Nutrition Survey (Encuesta Nacional de Salud y Nutrición; ENSANUT 2012) analyzed the food intake of 2057 children aged 0–47.9 months. What is notable about their diets is that low-nutrient-density cookies, sweet breads, and traditional beverages are consumed as the main source of food from the earliest days of life [23]. In the Netherlands, a dietary survey was conducted between 2011 and 2014 on 1526 children aged 10–48 months attending 199 daycare centers across the country. The study found that the preschoolers had higher than recommended energy and protein intakes, and some children exceeded the upper allowable intake limits for sodium, zinc, and retinol [24]. In the Flemish region of Belgium, 696 preschoolers aged 2.5–6.5 years were studied for sodium and potassium intake during 2002–2003. It was reported that more than 75% of children under 4 years of age and more than 40% of children over 4 years of age exceeded the Institute of Medicine (IOM) recommended upper limit for sodium, and more than three-quarters of children did not reach the IOM’s adequate intake for potassium [25].

Research from Korea and other countries has shown that early childhood dietary habits have a strong influence on childhood and adult dietary habits and health, so policies that promote healthy dietary habits at this age are needed. To develop such policies, we need scientific and objective data on the dietary habits of preschoolers. One area of research is the study of annual dietary trends. Numerous studies have examined the eating habits of Korean adults [26,27,28,29,30]. However, the only study analyzing the dietary habits of Korean preschool children by year has examined 3661 children aged 1–4 years, aimed at analyzing trends in growth and nutrient intake patterns in Korean toddlers and preschoolers [31]. The study used data from the 2010–2021 KNHANES, focusing on evaluating changes in growth status using the WHO 2006 child growth z-score standards. Additionally, a study analyzing dietary intake differences by gender among 3–5-year-old preschool children, based on 2013–2018 data from the KNHANES, has been reported [32]. The study found that boys had significantly higher intake of whole foods, grains, sugars, legumes, meat, eggs, dairy products, oils, and beverages than girls. It reported that both boys and girls had adequate energy and macronutrient intakes, but among micronutrients, potassium and calcium (only for girls) intake showed lower levels compared to the 2020 Korean Dietary Reference Intakes. However, these existing studies primarily focused on cross-sectional comparisons during specific survey periods or analyses centered on growth indicators, limiting their ability to provide a longitudinal perspective on how preschool children’s food and nutrient intakes and dietary habits have changed over time. Importantly, no annual dietary trend studies have been conducted for Korean preschool children, despite the recognized need for such research.

Therefore, the current study aimed to investigate food and nutrient intake and eating habit trends in children aged three to five years over the past ten years. To this end, we utilized data from 3- to 5-year-old preschool children who participated in the 2012–2020 National Health and Nutrition Examination Survey, which provides national dietary habit data. The results of the analysis are intended to provide guidelines for researchers studying early childhood nutrition and to inform appropriate dietary education.

## 2. Materials and Methods

### 2.1. Study Design and Population

Among the 77,834 KNHANES participants who completed a 24 h dietary recall survey from 2012 to 2021, subjects under 3 years of age or aged 5 years and older (n = 75,170) were excluded. Furthermore, subjects with total daily energy intake below the 1st percentile or exceeding the 99th percentile were excluded, applying the criteria from a previous study [21]. Additionally, participants lacking or having insufficient information on individual food and nutrient intake (n = 152) were excluded. Finally, participants who did not complete the dietary survey (n = 2) were also excluded, resulting in a final sample of 2510 participants (2012: n = 282, 2013: n = 313, 2014: n = 282, 2015 n = 209, 2016 n = 331, 2017 n = 278, 2018 n = 246, 2019 n = 236, 2020 n = 179, 2021 n = 154). This study was approved by the Institutional Review Board (IRB) of the Korea Disease Control and Prevention Agency <IRB approval numbers: 2007 KNHANES, 2007-02CON-04-P (approval date: 9 February 2007), 2008 KNHANES, 2008-04EXP-01-C (approval date: 14 February 2008), 2009 KNHANES, 2009-01CON-03-2C (approval date: 14 April 2009), 2010 KNHANES, 2010-02CON-21-C (approval date: 22 April 2010), 2011 KNHANES, 2011-02CON-06-C (approval date: 3 March 2011), 2012 KNHANES, 2012-01EXP-01-2C (approval date: 11 January 2012), 2013 KNHANES, 2013-07CON-03-4C (approval date: 16 July 2013), 2014 KNHANES, 2013-12EXP-03-5C (approval date: 31 December 2013), 2018 KNHANES, 2018-01-03-P-A (approval date: 12 January 2018); 2019 KNHANES, 2018-01-03-C-A (approval date: 19 December 2018); 2020 KNHANES, 2018-01-03-2C-A (approval date: 26 June 2020); 2021 KNHANES, 2018-01-03-5C-A (approval date: 23 April 2021)>. Among these, KNHANES was exempt from research ethics review under the Bioethics and Safety Act from 2015 to 2017. However, research ethics review was resumed from 2018, taking into account the provision of raw data to third parties and the collection of human specimens [33].

### 2.2. General Characteristics

General characteristics were analyzed such as gender, age, region, household income, mother’s age, and mother’s education level. The basic variables included in KNHANES were used without changes, such gender, age, region, and household income. Mother’s education level was appropriately modified for this study based on the health survey data. First, in terms of the level of education, subjects with elementary level or lower education and middle school graduates were integrated into the same category of “middle school or lower,” and the rest were classified into “high school” and “college or higher”.

### 2.3. Dietary Behavior

Dietary behavior was analyzed in terms of daily meal, type of meal according to eating place, eating-out frequency, and food security. To analyze the intake, daily meal (variable name: n_meal) was classified into breakfast, lunch, and dinner. Subjects who selected snacks (variable value: 4) were classified as having snacks and the rest as not having snacks. The eating-out frequency was used by modifying the questionnaire items from the dietary survey. The categories ‘2 or more times a day’ and ‘1 time a day’ were combined into ‘1 or more times a day,’ ‘5 to 6 times a week’ was used as is, and ‘1 to 2 times a week’ and ‘3 to 4 times a week’ were used as is. It was integrated into 1 to 4 times a week, and ‘1 to 3 times a month’ and ‘rarely (less than once a month)’ were reclassified as ‘less than once a week.’ Food security was classified based on the questionnaire item “Which of the following best describes the eating habits of the family over the past year?” which was newly introduced to KNHANES in 2005 [34,35]. The answer “enough foods of various types provided for every member of the family” was classified as enough food secure; “enough foods of not as various types provided for every member of the family” as mildly food insecure; and “insufficient foods provided due to financial difficulties” as severely food insecure.

### 2.4. Nutrient Intake

Daily nutrient intake (energy, carbohydrate, protein, fat, iron, sodium, calcium, phosphorus, potassium, thiamine, riboflavin, niacin, and vitamin C) were calculated using 24 h dietary recall data from the KNHANES. However, vitamin A intake was calculated as retinol equivalents (RE: retinol + 1/6 × beta-carotene) until the Sixth KNHANES (2013–2015), when the unit of evaluation for vitamin A in the Dietary Reference Intakes for Koreans was changed to retinol activity equivalents (RAE: Retinol + 1/12 × Beta-carotene), so from the raw data of the 2019 KNHANES, retinol activity equivalents (variable name: NF_VA_RAE) are provided [36,37]. Therefore, in this study, the retinol active equivalent variable was used for vitamin A intake for the integrated nutrient analysis of all data.

### 2.5. Food Group Intake

To analyze the food group intake of Korean young children, dietary survey data included in individual 24 h recall data were used. Food group classification was classified as follows using KNHANES’ food group classification (variable name: N_KINDG1): (1) Cereals and their products, (2) Potatos and starches, (3) Sugars and their products, (4) Beans or their products, (5) Nuts and their products, (6) Vegetables, (7) Mushrooms, (8) Fruits, (9) Seaweed, (10) Oils and fats, (11) Meat and their products, (12) Eggs, (13) Fish and shellfish, (14) Dairy products and their processed products, (15) Beverages, (16) Other foods.

### 2.6. Dish Group Intake

The classification by dish group (cooking method) was based on the dish group classification code (variable name: N_dcode) in the Nutrition Survey Codebook of the KNHANES and the classification used in previous studies [30,38]. ‘Rice,’ ‘Bread & snack (excluding some bread types such as cream bread and sweet red bean bread),’ ‘Noodle & dumpling,’ ‘Porridge,’ ‘Soup,’ ‘Stew/Hot pot foods,’ ‘Steamed foods,’ ‘Grilled foods,’ ‘Pan-fried foods,’ ‘Stir-fried foods,’ ‘Braised foods,’ ‘Fried foods,’ ‘Seasoned vegetables,’ ‘Seasoned fresh vegetables,’ ‘Kimchi,’ ‘Pickled/Preserved foods,’ ‘Seasoning,’ ‘Dairy products & ice creams,’ ‘Beverages,’ ‘Fruits,’ ‘Sweets (including honey, syrups, jams, gum, candy, chocolate, etc.),’ ‘Grain & potatoes (including some bakery products: Sweet red bean bread, cream bread, etc.),’ ‘Pulses, nuts & seeds,’ ‘Vegetables & seaweeds (raw vegetables: cucumber, Chinese cabbage, cabbage, seaweeds: wakame, nori, kelp, etc.),’ ‘Fishes & meat (fish cakes, sausages. ham, etc.),’ ‘Other dish group (butter, mayonnaise, sesame oil, salted fish, baby food, etc.).’

### 2.7. Statistical Analysis

All analyses were performed using SAS (Statistical Analysis System, SAS Institute, Cary, NC, USA) ver. 9.4, and the statistical significance level was set at α = 0.05. Since the KNHANES data are based on multi-stage stratified cluster sampling, analysis was performed considering the stratification variable (Kstrata), cluster variable (PSU, Primary Sampling Unit), and weight variable (Wt_ntr). Categorical variables such as the general characteristics and dietary behavior of the survey subjects were expressed as frequency (n) and weighted percentage (Weighted %) using Frequency Analysis, and significance was tested using chi-square. Continuous variables such as nutrient, food group, and dish group intake were expressed as mean and standard error using descriptive analysis. To evaluate the effect of the pandemic on dietary habits, we conducted significance tests on nutrient, food group, and dish group intake by year. As in the previous study [39], we used PROC SURVEYREG to calculate the *p* for trend and compare the estimated changes from eight years before the pandemic (2012–2019) and ten years from 2012 to the pandemic period (2021). We used the following adjustment variables: preschoolers’ gender, age, household income level, energy intake, mother’s age, and mother’s education.

## 3. Results

### 3.1. General Characteristics

The results of the general characteristics of Korean preschoolers by year are shown in Table 1. In terms of gender, in 2012, boys were 7.18% lower than girls, but in 2013 and later years, boys were over 50%, while girls were all between 43% and 49%, with no significant difference. Results for preschooler age, region, household income, height, weight, and BMI also showed no significant differences. However, mother’s age significantly increased by about 2.34 years over the past 10 years (*p* for trend < 0.001). In the case of mother’s education level, the proportion of college degree or higher significantly increased by about 10 percentage points over the past 10 years from 60.28% in 2012 to 70.35% in 2021 (*p* < 0.001).

### 3.2. Dietary Behaviors

The results of Korean young children’s dietary behavior by year are shown in Table 2. For snack consumption, the proportion of preschoolers eating snacks ranged from 96 to 99 percent from 2012 to 2021, although there was no significant difference. In terms of daily meal trends, there was no significant difference for breakfast, but the rate of skipping meals decreased by 5.46 percentage points over the past decade, from 14.61% in 2012 to 9.15% in 2021. Regarding frequency of eating out, five to six times per week was the highest rate from 2012 to 2021. Eating out at least once a day tended to increase by approximately 30 percentage points over the past 10 years, from 15.17% in 2012 to 45.65% in 2021. In terms of food security, the prevalence of enough food security tended to increase by about 27.28 percentage points over the past 10 years, from 45.60% in 2012 to 72.88% in 2021, while the prevalence of moderate/severe food insecurity decreased by about 5.95 percentage points over the past 10 years, from 6.56% in 2012 to 0.61% in 2021.

### 3.3. Nutrient Intake

Table 3 shows the trends in nutrient intake among Korean preschool children by year. There was no significant difference in energy, vitamin A, and calcium intake over the past decade. Among the nutrients that provide energy, carbohydrate intake decreased until 2021 (unadjusted *p* for trend < 0.05, adjusted *p* for trend < 0.001), while protein and fat intake increased over the past decade (adjusted *p* for trend < 0.01). The carbohydrate contribution rate decreased, while the contribution rates of protein and fat showed an increasing trend over the past decade. Among minerals, intakes of iron, sodium, and potassium showed an overall decreasing trend over the past decade (unadjusted *p* for trend < 0.001, adjusted *p* for trend < 0.001). Phosphorus intake showed no significant trend until 2019, before the onset of the pandemic, but showed a significant decreasing trend during the pandemic (adjusted *p* for trend < 0.001). Carotene, thiamine, niacin, and vitamin C intake decreased significantly from 2012 to 2019 and during the pandemic period through 2021 (adjusted *p* for trend < 0.01). Conversely, retinol and riboflavin intake increased over the past decade, from 2012 to 2021 (unadjusted *p* for trend < 0.001, adjusted *p* for trend < 0.001).

### 3.4. Food Group Intake

Table 4 shows the food group intake of Korean preschoolers. The intake of the following food groups increased from 2012 to 2021: ‘Sugar and their products,’ ‘Meat and their products,’ ‘Eggs and their products,’ ‘Fish and shellfish,’ ‘Beverages,’ and ‘Other foods’ (unadjusted *p* for trend < 0.05, adjusted *p* for trend < 0.01). Intake of ‘Grain and cereal products’ and ‘nuts and seed products’ decreased significantly from 2012 to 2019, prior to the onset of the pandemic. However, there was no significant difference in intake trends from 2012 to 2021 during the pandemic. Fruit intake showed no significant trend change from 2012 to 2019, before the pandemic, but a significant decrease from 2012 to 2021 during the pandemic (unadjusted *p* for trend < 0.01, adjusted *p* for trend < 0.01).

### 3.5. Daily Food Intake by Dish Group

Table 5 shows the daily food intake of Korean preschoolers by dish group. The intake of ‘Noodles & Dumplings,’ ‘Soup,’ ‘Grilled foods,’ and ‘Sweets’ has increased over the past decade (unadjusted *p* for trend < 0.05, adjusted *p* for trend < 0.05). In contrast, intake of ‘Fruits’ and ‘Pickled/Preserved foods’ did not differ significantly from 2012 to 2019, before the pandemic, but decreased significantly from 2012 to 2021 (adjusted *p* for trend < 0.05). There was a significant increase in the intake of ‘Pan-fried foods’ from 2012 to 2019, before the pandemic, and a non-significant trend in intake from 2012 to 2021, during the pandemic.

### 3.6. Foods Contributing to the Total Food Intake

The top 20 food sources of total food intake from 2012 to 2021 are shown in Table 6. Milk, white rice/glutinous rice, apples, and eggs were the most common foods in the top 10 from 2012 to 2021. Of these, milk, the number one food, contributed between 15 and 22 percent to total food intake from 2012 to 2021, followed by white rice/glutinous rice, which contributed between 9.5 and 12.0 percent to total food intake. Eggs and apples fluctuated in the rankings but contributed between 2 and 7 percent to total food intake. Looking at the top 10 food sources that contributed to total food intake in 2021 during the COVID-19 pandemic, milk (18.86%), white/non-glutinous rice (10.46%), fish broth (4.66%), apples (3.35%), eggs (3.14%), fruit drinks (2.95%), pork (2.75%), kelp broth (2.08%), yogurt (2.04%), and tofu (1.75%) appeared in that order.

## 4. Discussion

This study analyzed the dietary trends of Korean preschoolers using KNHANES data from 2012 to 2021. The snack consumption rate was found to be over 96% for all 10 years. Because of previous studies, the increase in snack consumption was pointed out to be nutritionally problematic [40,41,42,43]. Therefore, it is deemed necessary to conduct specific research on changes in energy density and nutrient density according to the amount and type of snacks primarily consumed by children, like a previous study [44]. The breakfast skipping rate showed a tendency to decrease by 5.46 percentage points over the past 10 years, from 14.61% in 2012 to 9.15% in 2021. However, in all years, the breakfast skipping rate was found to be higher than the dinner or lunch skipping rate. A previous study by Kim, EK et al. [11] reported that preschool children attending childcare facilities may skip breakfast during the early-morning drop-off process. Furthermore, the study noted that skipping breakfast may occur when both parents work, as the simultaneous demands of preparing for work and taking children to childcare in the early morning may lead to skipping breakfast. In addition, the increase in skipping breakfast is pointed out as a major problem not only in preschoolers but also as the life cycle progresses from childhood to adolescence [30,45]. Regular breakfast is reported to have a positive effect on desirable eating habits, nutritional quality of meals [40,46], learning, and cognitive abilities [40,46,47]. Therefore, it is believed that it will be necessary to consider dietary characteristics according to the life cycle when developing dietary education programs related to skipping breakfast in the future.

The frequency of eating out (including institutional meals) among preschoolers showed that over 90% of preschool children were eating out five or more times per week from 2012 to 2021. Among these, the frequency of eating out once or more per day increased by approximately 30 percentage points, from 15.17% in 2012 to 45.65% in 2021. These results are like those mentioned in previous studies [11,32]. This high rate is presumed to be due to children attending childcare facilities and consuming meals provided there. However, the KNHANES’ eating-out frequency item includes not only meals provided at childcare facilities but also fast food and commercial eating out. Therefore, the high frequency of eating out appears to warrant attention. Reviewing a study evaluating the quality of meals consumed by Korean infants, toddlers, and school-age children through eating out, comparisons of two eating-out patterns (rice-centered and mixed) reported deficiencies in both quantity and quality [48]. To address these issues, academia and research institutions should consider studies such as evaluating the quality of meals provided to preschool children by location [49,50,51]. Furthermore, the industry needs to develop children’s menus that consider the quality of meals at eating-out locations, while the government and local authorities should establish policy standards for this purpose.

**Figure 1:**
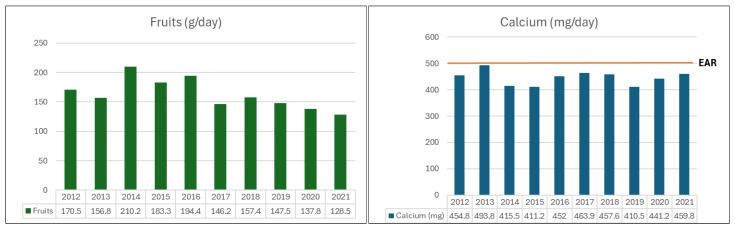
Fruit and calcium intake by year of Korean preschoolers.

Among the yearly nutritional intake results of this study, sodium intake in this study showed a decreasing trend over the past 10 years from 2012 to 2021. However, sodium intake among preschool children aged 3–5 years was found to exceed 1600 mg, the intake standard for reducing chronic disease risk first established in the 2020 KDRIs [52]. Soup-based dishes—a major source of sodium—are managed through the Korean center for children’s foodservice management to ensure they are prepared at or below the recommended salt level of 0.5% for children [48]. However, in the case of soup, one of the leading acute sources of sodium, while children’s cafeterias are regulated by the Korean Center for Children’s Foodservice Management to prepare soup at or below the recommended 0.5% salt level for children, commercial locations are not regulated to prepare soup at a separate salt level for children, which can lead to excess sodium intake in children as well as adults [48,53]. Therefore, preschool-aged children, whose adequate sodium intake levels are lower than adults’, are particularly vulnerable to excessive sodium intake when eating out, especially outside of group meals provided by childcare facilities [48]. Based on previous studies and the results of this present study, it is suggested that future policy measures should be developed to improve these issues. Meanwhile, calcium intake of this study was found to be more than 100 mg lower than the average requirement of 500 mg in all years [52]. Meanwhile, this present study found that calcium intake was at least 100 mg lower than the estimate average requirement of 500 mg in all years (Figure 1). In case of milk, a primary source of calcium [54], this present study ranked first or second in total food consumption. However, despite high milk consumption, calcium intake remained low, suggesting the need for measures to improve calcium intake. This is because calcium is a micronutrient necessary for skeletal growth in preschoolers and regulates various physiological functions such as blood clotting, nerve transmission, muscle contraction and relaxation, and cell metabolism. Additionally, potassium is present in all tissues of the body and is an essential mineral for normal cell functions, such as intracellular fluid volume [52]. Attention should be paid to ensuring adequate calcium intake during childhood, as insufficient intake at this stage lowers peak bone mass during growth. This increases the risk of developing osteopenia and osteoporosis when bone loss occurs in adulthood [55]. Based on these previous studies and the results of this present study, it is considered necessary to develop dietary education programs for children aimed at balanced nutrient intake, along with guidelines for balanced diets.

In this study, fruit consumption among food groups was found to have decreased by approximately 42.0 g over the past 10 years (Figure 1). The importance of fruit intake has already been introduced in many studies, and fruit and vegetable intake have been reported to play an important role as a source of various vitamins and minerals involved in energy metabolism for growing children with high-energy requirements [1]. In addition, it has been reported that adequate fruit consumption is effective in preventing various chronic degenerative diseases such as high blood pressure, diabetes, and cancer after adulthood [56,57,58]. Additionally, if fruit intake continues to decrease with age, it may also affect the onset and progression of diet-related diseases [56]. It is believed that continuous monitoring and supplementary measures for preschoolers’ dietary habits are necessary to ensure balanced and appropriate intake of plant-based foods, including fruits.

This study has several limitations. First, dietary intake data were collected using a 24 h recall method, which may not accurately reflect participants’ usual intake [31,59,60]. Additionally, since dietary information was obtained from primary caregivers, it may not fully capture meals consumed outside the home, such as at kindergartens or daycare centers. Therefore, the data may not fully reflect children’s total food intake [31]. Second, the pandemic may have affected the KNHANES survey. Previous studies have shown that, in 2012, 2019, and 2021, all sampled wards completed the survey. However, in 2020, only 166 of 192 wards (86%) conducted dietary surveys due to the pandemic. Nevertheless, no significant differences were observed in participants’ gender, age, or income level between 2019 and 2020. Additionally, food and nutrient intake among Korean adults was reported to have remained stable between 2019 and 2020, despite the pandemic [33,39]. It will be necessary, however, to monitor whether the pandemic affects dietary intake, health, and quality of life in the long term through future follow-ups, such as cohort data collection. Finally, since this study aimed to analyze the intake of ‘dietary behaviors,’ ‘food groups,’ ‘nutrients,’ and ‘food groups’ and ’foods contributing to the total food’ by year to assess dietary habits by year, it was not possible to analyze dietary patterns. It is recommended that future studies should be conducted to analyze changes in dietary patterns by year.

Despite these limitations, this study is significant because it used nationally representative data to examine long-term trends in dietary behavior and food and nutrient intake among Korean preschoolers. Additionally, since this study examined the dietary trends of Korean preschool children, it is expected to be used in the future to develop dietary/nutrition education content or guidelines for each life cycle.

## 5. Conclusions

This study analyzed changes in the dietary intake and eating behaviors of Korean children aged three to five years (n = 2510) based on data from the 2012–2021 KNHANES. This study drew the following conclusions. First, the rate of breakfast skipping decreased from 14.61% in 2012 to 9.15% in 2021. Meanwhile, the frequency of eating out at least once a day (including institutional meals) increased by about 30 percentage points, rising from 15.17% in 2012 to 45.65% in 2021. Second, changes in energy sources and nutrient intake were observed: a significant decrease in carbohydrate, phosphorus, iron, potassium, carotene, niacin, and vitamin C, as well as a significant decrease in carbohydrate energy ratios; and a significant increase in protein, fat, retinol, thiamine, riboflavin, and protein-fat energy ratios. Third, intake of ‘Sugar and their products,’ ‘Meat and their products,’ ‘Eggs and their products,’ ‘Fish and shellfish,’ and ‘Beverages’ increased by food group, while intake of ‘Grain and cereal products,’ ‘Nuts and seed products,’ and ‘fruits’ decreased, especially fruit intake, which decreased by about 42.0 g. Fourth, the nutrient intake assessment showed that calcium intake was approximately 100 mg lower than the estimated average requirement of 500 mg in all years. Sodium intake decreased but still exceeded the level recommended for reducing chronic disease risk (1600 mg). Therefore, in order to manage preschoolers’ dietary habits, including balanced food and nutrient intake, it is necessary to establish a continuous monitoring system of children’s lunch menus in childcare facilities, develop customized dietary education programs, and establish dietary guidelines to increase the intake of plant foods including fruits and nutrients such as calcium. This study could serve as the basis for future preschool dietary policies and educational guidelines.

## Figures and Tables

**Table 1:** General characteristics by survey year of Korean preschoolers.

Variables	2012 (n = 282)		2013 (n = 313)		2014 (n = 282)		2015 (n = 209)		2016 (n = 331)		2017 (n = 278)		2018 (n = 246)		2019 (n = 236)		2020 (n = 179)		2021 (n = 154)		***p*** **-Value ^(1)^**
	n	%	n	%	n	%	n	%	N	%	n	%	n	%	n	%	n	%	n	%	
Gender																					
Boy	150	46.41	159	51.96	156	53.44	110	54.74	165	51.39	154	52.13	126	56.28	128	53.51	93	53.57	79	51.88	0.8591
Girl	132	53.59	154	48.04	126	46.56	99	45.26	166	48.61	124	47.87	120	43.72	108	46.49	86	46.43	75	48.12	
Age (years)																					
3	92	34.22	103	33.52	99	34.93	73	38.21	115	33.80	84	27.97	80	33.60	84	36.56	55	28.62	45	31.01	0.6641
4	87	32.12	104	33.72	96	33.49	63	28.98	114	33.82	100	37.96	74	29.75	78	34.45	63	36.82	38	29.01	
5	103	33.65	106	32.76	87	31.57	73	32.81	102	32.38	94	34.07	92	36.64	74	28.99	61	34.55	71	39.98	
Mean age	3.99	0.06	3.99	0.05	3.97	0.05	3.95	0.06	3.99	0.05	4.06	0.05	4.03	0.06	3.92	0.06	4.06	0.06	4.09	0.07	0.7441(+) ^(2)^
Region																					
City	245	84.27	257	80.09	242	86.24	181	86.95	268	83.10	235	85.83	217	89.61	200	87.52	151	84.43	127	86.16	0.9455
Rural area	37	15.73	56	19.91	40	13.76	28	13.05	63	16.90	43	14.17	29	10.39	36	12.48	28	15.57	27	13.84	
Household income																					
Low	18	8.05	27	8.83	17	6.13	15	6.37	20	6.65	17	5.90	22	10.26	11	4.48	14	7.17	5	2.86	0.7501
Middle–low	82	34.05	102	32.11	81	29.08	55	29.01	105	34.93	86	30.95	73	34.56	82	34.08	49	24.94	51	33.85	
Middle–high	100	31.97	101	32.55	111	40.31	77	36.65	116	33.92	97	37.00	88	32.37	90	37.79	56	32.57	51	36.63	
High	72	25.93	82	26.52	73	24.48	61	27.97	88	24.50	78	26.15	63	22.81	53	23.65	60	35.32	47	26.66	
Height (cm)	105.29	0.63	105.43	0.40	105.32	0.47	105.57	0.48	105.82	0.49	106.29	0.52	106.02	0.47	106.28	0.53	106.61	0.70	106.55	0.66	0.1015(+) ^(2)^
Weight (kg)	17.58	0.32	17.60	0.19	17.60	0.22	17.51	0.20	17.82	0.23	17.98	0.25	17.66	0.21	18.13	0.25	18.66	0.36	17.82	0.28	0.0999(+) ^(2)^
BMI ^(2)^ (kg/m^2^)	15.74	0.14	15.75	0.11	15.76	0.10	15.61	0.10	15.81	0.10	15.83	0.10	15.62	0.12	15.94	0.11	16.27	0.16	15.60	0.12	0.2984(+) ^(2)^
Mother’s characteristics																					
Mother’s age (years)	34.36	0.45	35.07	0.33	35.04	0.27	35.32	0.32	35.57	0.25	36.25	0.30	36.34	0.36	36.35	0.29	36.93	0.41	36.70	0.53	<0.0001(+) ^(2)^
Mother’s education level																					
Less than high-school graduate	4	1.56	3	0.94	6	2.43	3	1.76	11	3.99	3	1.23	6	3.26	5	2.40	4	2.16	8	5.49	<0.0001
High-school diploma	80	38.16	84	33.16	73	30.51	50	28.66	67	26.48	41	18.59	41	21.40	27	14.78	26	16.18	26	24.16	
College degree or higher	149	60.28	178	65.90	161	67.05	124	69.58	208	69.53	185	80.17	175	75.34	186	82.82	118	81.66	103	70.35	

^(1)^ *p*-value by chi-square. ^(^^2)^ *p* for trend by Generalized Linear Model (GLM).

**Table 2:** Dietary behaviors by year of Korean preschoolers.

Variables	2012 (n = 282)		2013 (n = 313)		2014 (n = 282)		2015 (n = 209)		2016 (n = 331)		2017 (n = 278)		2018 (n = 246)		2019 (n = 235)		2020 (n = 179)		2021 (n = 154)		*p*-Value ^(1)^
	n	%	n	%	n	%	N	%	N	%	N	%	n	%	n	%	n	%	n	%	
Snack																					0.3077
Yes	272	96.35	308	98.41	278	98.63	203	96.84	325	98.18	275	98.87	245	99.58	232	98.37	173	97.54	152	99.43	
No	10	3.65	5	1.59	4	1.37	6	3.16	6	1.82	3	1.13	1	0.42	4	1.63	6	2.46	2	0.57	
Breakfast																					0.2419
Skipped	34	14.61	28	8.68	30	9.71	12	6.04	25	7.07	21	6.87	18	8.13	23	10.59	22	12.12	14	9.15	
Eaten	248	85.39	285	91.32	252	90.29	197	93.96	306	92.93	257	93.13	228	91.87	213	89.41	157	87.88	140	90.85	
Lunch																					0.2617
Skipped	11	3.43	10	2.67	11	3.81	4	1.62	18	6.46	10	4.45	13	6.38	9	3.98	6	3.11	4	2.44	
Eaten	271	96.57	303	97.33	271	96.19	205	98.38	313	93.54	268	95.55	233	93.62	227	96.02	173	96.89	150	97.56	
Dinner																					0.6182
Skipped	10	3.50	13	4.39	6	1.71	5	2.34	4	1.20	6	2.48	3	1.29	4	2.25	4	1.77	4	2.20	
Eaten	272	96.50	300	95.61	276	98.29	204	97.66	327	98.80	272	97.52	243	98.71	232	97.75	175	98.23	150	97.80	
Eating-out frequency																					<0.0001
≥1 time/day	41	15.17	81	25.13	118	41.31	79	36.64	108	31.81	87	34.65	100	34.47	86	39.65	70	38.58	71	45.65	
5–6 times/week	221	79.50	214	68.60	155	55.23	120	58.69	209	63.63	169	58.36	139	62.52	144	58.19	82	46.10	71	45.91	
1–4 times/week	13	3.62	13	4.24	7	2.94	9	4.16	9	2.71	19	5.87	5	2.44	4	1.61	26	14.59	10	6.84	
<1 times/week	7	1.72	5	2.03	2	0.52	1	0.51	5	1.86	3	1.12	2	0.58	2	0.56	1	0.72	2	1.61	
Food security																					<0.0001
Enough food secure	133	45.60	149	45.27	145	49.58	119	55.44	184	56.40	189	69.21	164	67.35	156	68.09	123	70.86	115	72.88	
Mild food insecure	134	47.85	153	51.58	130	46.92	85	42.54	140	40.67	83	28.78	78	30.45	77	31.05	52	27.19	37	26.51	
Moderate/severe food insecure	13	6.56	10	3.15	7	3.51	5	2.02	7	2.93	6	2.01	4	2.20	2	0.86	4	1.95	2	0.61	

^(1)^ *p*-value by chi-square.

**Table 3:** Nutrient intake trends by year in Korean preschoolers.

Nutrient	2012 (n = 282)		2013 (n = 313)		2014 (n = 282)		2015 (n = 209)		2016 (n = 331)		2017 (n = 278)		2018 (n = 246)		2019 (n = 235)		2020 (n = 179)		2021 (n = 154)		KNHANES 2012~2019		KNHANES 2012~2021	
	Mean	SE	Mean	SE	Mean	SE	Mean	SE	Mean	SE	Mean	SE	Mean	SE	Mean	SE	Mean	SE	Mean	SE	Unadjusted *p* for Trend ^(1)^	Adjusted *p* for Trend ^(1), (2)^	Unadjusted *p* for Trend ^(1)^	Adjusted *p* for Trend ^(1), (2)^
Energy (kcal)	1293.4	39.3	1441.6	64.9	1375.6	28.0	1383.2	36.2	1375.9	34.1	1391.9	30.0	1321.2	29.2	1336.1	34.5	1435.0	51.2	1370.3	35.1	0.5538(−)	0.1812(−)	0.7243(−)	0.3048(+)
Carbohydrate (g)	208.6	6.9	220.5	8.6	218.4	4.9	221.6	5.9	218.3	4.9	218.6	3.8	205.3	4.7	202.0	5.0	212.0	7.0	204.4	5.8	0.0835(−)	0.0023(−)	0.0234(−)	<0.001(−)
Protein (g)	43.5	1.4	46.3	1.9	45.3	1.1	44.7	1.4	45.7	1.3	49.7	1.7	45.7	1.3	46.8	1.5	50.0	2.1	48.8	1.8	0.2792(+)	0.0090(+)	0.0746(+)	0.0020(+)
Fat (g)	32.0	1.3	40.4	3.2	35.0	1.1	34.2	1.2	35.0	1.4	39.4	1.8	34.5	1.3	37.2	1.4	42.3	2.3	38.8	1.4	0.6544(+)	0.0907(+)	0.1004(+)	0.0003(+)
Calcium (mg)	454.8	23.1	493.8	45.5	415.5	15.0	411.2	15.9	452.0	18.5	463.9	17.9	457.6	18.1	410.5	17.1	441.2	24.0	459.8	21.7	0.4020(−)	0.7737(−)	0.4703(−)	0.6404(−)
Phosphorus (mg)	797.0	23.9	838.5	66.3	760.4	18.4	757.2	23.4	753.7	21.7	806.8	27.2	750.2	19.2	736.0	23.5	781.7	33.9	787.2	28.5	0.0590(−)	0.0511(−)	0.1190(−)	<0.001(−)
Iron (mg)	7.7	0.5	9.3	0.6	9.2	0.3	9.2	0.4	7.3	0.3	8.1	0.3	6.9	0.2	5.5	0.2	5.8	0.3	5.9	0.5	<0.001(−)	<0.001(−)	<0.001(−)	<0.001(−)
Sodium (mg)	1945.5	108.2	1851.6	115.1	1756.9	61.0	1836.3	66.9	1638.8	63.1	1719.3	50.0	1535.4	51.9	1629.2	62.5	1730.3	93.4	1630.4	77.6	<0.001(−)	<0.001(−)	<0.001(−)	<0.001(−)
Potassium (mg)	1727.7	61.5	1818.3	47.8	1903.9	50.4	1850.5	60.7	1743.5	50.5	1768.0	43.3	1671.0	50.2	1591.3	48.4	1667.8	73.5	1610.4	58.5	0.0010(−)	0.0012(−)	<0.001(−)	<0.001(−)
Vitamin A (μg RAE)	470.4	36.9	439.2	33.0	495.1	43.3	456.7	29.9	451.6	25.0	296.7	17.6	430.9	23.3	281.2	14.4	304.0	22.9	341.4	19.9	0.2726(+)	0.1440(+)	0.3659(+)	0.2764(+)
Carotene (μg)	1892.9	195.6	1479.8	119.4	2073.5	241.2	1791.8	162.7	1386.4	92.9	1400.3	87.1	1284.2	99.2	1208.2	83.5	1390.4	143.9	1190.0	116.8	<0.001(−)	<0.001(−)	<0.001(−)	<0.001(−)
Retinol (μg)	139.7	8.7	162.9	16.3	119.0	6.0	134.0	8.4	220.6	20.7	251.2	22.2	216.9	17.9	180.5	10.9	188.2	17.1	242.2	18.1	<0.001(+)	<0.001(+)	<0.001(+)	<0.001(+)
Thiamin (mg)	0.8	0.03	1.3	0.04	1.2	0.04	1.2	0.05	0.9	0.04	1.0	0.04	0.9	0.04	0.8	0.0	0.8	0.0	0.9	0.0	<0.001(−)	<0.001(−)	<0.001(−)	<0.001(−)
Riboflavin (mg)	1.0	0.04	1.1	0.05	1.0	0.03	1.0	0.04	1.2	0.05	1.3	0.1	1.2	0.04	1.1	0.0	1.3	0.1	1.3	0.1	0.0003(+)	<0.001(+)	<0.001(+)	<0.001(+)
Niacin (mg)	8.5	0.3	8.9	0.5	9.5	0.3	8.8	0.3	7.7	0.3	8.4	0.3	7.8	0.3	7.5	0.3	8.2	0.5	8.7	0.5	0.0002(−)	<0.001(−)	0.0063(−)	0.0005(−)
Vitamin C (mg)	72.8	5.9	64.2	4.2	84.7	7.9	76.7	7.6	58.2	3.9	63.7	5.5	54.7	4.1	63.6	4.9	57.7	5.3	60.8	6.7	0.0052(−)	0.0078(−)	0.0025(−)	0.0060(−)
Energy contribution (%)																								
Carbohydrate (%)	64.4	0.8	63.2	0.5	64.0	0.6	65.1	0.6	64.5	0.5	61.5	0.7	63.0	0.7	61.4	0.6	60.4	0.8	60.7	0.7	0.0069(−)	0.0067(−)	<0.001(−)	<0.001(−)
Protein (%)	13.5	0.3	13.1	0.2	13.2	0.2	12.9	0.2	13.4	0.2	14.1	0.2	13.8	0.3	14.0	0.2	13.9	0.2	14.1	0.3	0.0086(+)	0.0091(+)	0.0022(+)	0.0035(+)
Fat (%)	22.1	0.6	23.7	0.5	22.7	0.5	22.0	0.4	22.2	0.4	24.4	0.6	23.2	0.5	24.6	0.5	25.8	0.7	25.3	0.6	0.0397(+)	0.0272(+)	<0.001(+)	<0.001(+)

^(^^1)^ *p* for trend by Generalized Linear Model (GLM), ^(^^2)^ Adjusted for gender, age, mother’s age, mother’s education level and energy intake.

**Table 4:** Trends in food intake by year of Korean preschoolers.

Food Group (g/day)	2012 (n = 282)		2013 (n = 313)		2014 (n = 282)		2015 (n = 209)		2016 (n = 331)		2017 (n = 278)		2018 (n = 246)		2019 (n = 235)		2020 (n = 179)		2021 (n = 154)		KNHANES 2012~2019		KNHANES 2012~2021	
	Mean	SE	Mean	SE	Mean	SE	Mean	SE	Mean	SE	Mean	SE	Mean	SE	Mean	SE	Mean	SE	Mean	SE	Unadjusted *p* for Trend ^(1)^	Adjusted *p* for Trend ^(1), (2),^	Unadjusted *p* for Trend ^(1)^	Adjusted *p* for Trend ^(1), (2)^
Total food	929.7	25.3	984.7	24.4	1010.8	28.0	1015.0	31.4	1006.1	27.8	1036.7	25.1	976.4	25.2	963.9	27.9	1033.3	32.4	998.5	36.7	0.5638(+)	0.1803(+)	0.5562(+)	0.3381(+)
Grain and cereal products	202.7	10.0	214.6	12.8	202.5	6.8	215.4	6.8	207.4	6.5	207.1	5.3	181.5	6.5	198.1	6.3	216.0	11.1	201.7	8.4	0.0281(−)	0.0036(−)	0.2754(−)	0.2487(+)
Potatoes and starch products	22.8	3.8	27.1	3.7	30.5	3.9	23.5	4.1	23.4	2.5	19.3	2.8	25.8	5.2	22.1	3.4	22.7	3.9	25.0	4.1	0.2820(−)	0.4008(−)	0.4934(−)	0.6178(−)
Sugar and their products	8.8	1.5	8.1	1.1	10.5	1.6	10.3	1.5	8.0	1.0	13.2	2.1	11.3	1.4	13.3	1.6	12.1	1.7	12.4	1.8	0.0116(+)	0.0062(+)	0.0026(+)	0.0008(+)
Beans and their products	24.3	4.9	19.5	2.6	22.0	3.0	20.8	3.7	23.5	4.4	27.5	4.3	19.2	3.0	16.9	3.2	12.9	2.9	29.2	5.9	0.3158(−)	0.2335(−)	0.5789(−)	0.4556(−)
Nuts and seeds products	2.1	0.5	1.6	0.2	4.8	1.5	3.1	0.9	3.3	1.2	2.0	0.4	1.7	0.3	1.9	0.4	1.3	0.3	2.3	0.6	0.0434(−)	0.0317(−)	0.0843(−)	0.0780(−)
Vegetables	90.4	8.5	89.8	5.0	98.2	4.5	105.1	6.5	93.9	4.7	94.2	5.5	92.8	6.3	89.8	5.6	97.2	8.1	76.7	7.4	0.7909(−)	0.9225(−)	0.2454(−)	0.2283(−)
Mushrooms	2.8	0.6	3.4	0.7	3.5	0.6	3.5	0.7	3.6	0.5	4.7	0.7	4.3	0.9	3.9	0.7	3.9	0.8	3.4	0.8	0.0621(+)	0.0511(+)	0.1256(+)	0.0887(+)
Fruits	170.5	15.9	156.8	12.6	210.2	18.0	183.3	17.5	194.4	14.3	146.2	11.7	157.4	15.3	147.5	15.7	137.8	13.1	128.5	14.8	0.1762(−)	0.1396(−)	0.0010(−)	0.0005(−)
Seaweeds	3.2	0.7	8.0	2.0	10.4	2.3	12.2	2.8	12.8	2.3	19.1	2.6	24.4	4.6	20.2	4.4	23.3	5.7	24.5	5.8	<0.0001(+)	<0.0001(+)	<0.0001(+)	<0.0001(+)
Oil and fat	3.9	0.4	4.3	0.4	4.5	0.4	4.8	0.5	4.3	0.4	5.2	0.5	4.5	0.3	4.6	0.4	5.1	0.9	5.0	0.5	0.3157(+)	0.2526(+)	0.4034(+)	0.4269(+)
Meat and their products	50.4	4.9	54.3	4.1	58.5	4.1	50.8	5.1	53.7	4.1	67.4	5.1	59.5	4.5	74.7	5.7	83.1	5.4	65.2	5.6	0.0028(+)	0.0016(+)	<0.0001(+)	<0.0001(+)
Eggs and their products	23.8	3.2	27.1	2.5	25.6	2.3	30.5	2.5	28.2	2.2	35.0	8.6	26.8	2.6	33.7	3.1	31.9	3.5	31.8	3.1	0.0199(+)	0.0074(+)	0.0088(+)	0.0055(+)
Fish and shellfish	19.7	2.5	27.4	4.1	34.1	4.1	37.4	4.7	41.3	5.4	47.6	4.2	48.2	4.6	46.7	6.0	43.3	7.5	68.8	13.4	<0.0001(+)	<0.0001(+)	<0.0001(+)	<0.0001(+)
Milk and dairy products	255.3	21.3	268.9	14.4	220.6	12.6	220.6	13.1	234.8	14.9	241.6	14.1	255.9	17.0	202.5	13.3	222.4	17.6	248.3	17.8	0.1756(−)	0.2882(−)	0.2076(−)	0.2216(−)
Beverages	35.0	6.7	58.9	7.2	58.6	6.7	73.0	9.1	58.2	5.9	89.7	10.2	46.1	6.4	72.7	9.7	101.3	13.7	55.8	10.2	0.0499(+)	0.0449(+)	0.0102(+)	0.0053(+)
Other food	13.8	1.3	14.7	1.7	16.4	1.7	20.5	2.9	15.4	1.4	17.0	1.7	22.1	3.1	15.1	1.5	19.0	1.7	19.7	2.0	0.0013(+)	0.0013(+)	0.0047(+)	0.0063(+)

^(1)^ *p* for trend by Generalized Linear Model (GLM), ^(2)^ Adjusted for gender, age, mother’s age, mother’s education level, and energy intake.

**Table 5:** Trend in daily food intake by dish group of Korean preschoolers.

Dish Group (g/day)	2012 (n = 282)		2013 (n = 313)		2014 (n = 282)		2015 (n = 209)		2016 (n = 331)		2017 (n = 278)		2018 (n = 246)		2019 (n = 235)		2020 (n = 179)		2021 (n = 154)		KNHANES 2012~2019		**KNHANES 2012~2021**	
	Mean	SE	Mean	SE	Mean	SE	Mean	SE	Mean	SE	Mean	SE	Mean	SE	Mean	SE	Mean	SE	Mean	SE	Unadjusted *p* for Trend ^(1)^	**Adjusted** ***p*** **for Trend ^(2)^**	**Unadjusted** ***p*** **for Trend ^(1)^**	**Adjusted** ***p*** **for Trend ^(2)^**
Rice	135.3	6.4	147.4	12.2	144.2	6.2	162.6	9.0	139.1	5.4	158.8	10.5	139.9	6.5	142.4	5.8	147.2	8.7	144.8	8.4	0.6653(−)	0.6949(+)	0.6090(−)	0.9573(−)
Bread & snack	41.4	4.5	63.2	12.6	43.6	3.9	43.0	3.8	51.2	4.4	42.5	3.3	42.8	4.3	43.7	3.7	48.6	5.3	33.0	4.3	0.3027(−)	0.3320(−)	0.1464(−)	0.1408(−)
Noodle & dumpling	27.2	4.7	37.7	5.8	36.5	5.7	38.7	6.6	53.8	7.4	50.5	7.4	36.7	5.7	44.5	5.5	58.1	11.1	40.9	9.7	0.0226(+)	0.0336(+)	0.0159(+)	0.0301(+)
Porridge	15.8	3.2	11.2	2.0	16.1	3.7	16.5	2.7	11.2	2.4	8.9	1.9	18.7	5.3	11.6	2.5	12.7	3.1	17.3	5.7	0.4763(−)	0.5613(−)	0.9083(−)	0.8752(+)
Soup	40.4	6.0	38.1	5.1	47.9	5.6	50.3	7.6	53.4	5.6	64.9	6.5	64.3	8.0	71.2	10.3	72.0	10.4	84.5	21.4	<0.001(+)	0.0002(+)	0.0015(+)	0.0037(+)
Stew/hot pot foods	9.2	1.7	13.9	2.2	17.8	3.3	13.8	3.0	19.4	3.8	17.2	3.6	15.0	4.6	12.5	2.7	5.0	1.3	15.6	4.1	0.3921(+)	0.2313(+)	0.7643(−)	0.7889(+)
Steamed foods	9.1	2.3	6.7	1.4	11.6	1.9	11.4	3.1	9.8	2.5	9.6	1.9	7.1	1.3	10.8	2.7	11.3	4.3	7.1	1.8	0.8316(−)	0.7199(−)	0.1358(−)	0.0981(−)
Grilled foods	18.6	2.8	25.6	2.4	26.2	3.0	22.8	3.2	23.0	2.4	31.2	3.8	33.0	4.4	31.8	3.5	35.4	4.8	36.7	5.9	0.0007(+)	0.0013(+)	<0.0001(+)	<0.0001(+)
Pan-fried foods	21.5	3.0	21.0	2.4	21.0	2.5	23.4	2.9	20.2	2.5	23.8	3.4	27.0	3.7	27.0	4.1	24.8	3.5	26.0	3.8	0.0499(+)	0.0278 (+)	0.0700(+)	0.0508(+)
Stir-fried foods	22.3	2.5	16.5	1.9	19.8	2.3	24.1	7.0	20.1	2.1	19.7	2.5	19.6	2.5	26.3	5.3	20.8	3.3	22.4	3.3	0.6115(+)	0.5314(+)	0.5553(+)	0.4200(+)
Braised foods	11.0	2.4	13.5	3.0	8.5	1.4	9.5	1.7	11.1	1.9	13.5	2.6	12.8	2.3	11.1	2.1	12.4	2.6	17.7	3.7	0.3230(+)	0.1799(+)	0.0758(+)	0.0391(+)
Fried foods	21.1	4.5	19.5	4.3	24.2	3.5	21.2	4.0	18.3	3.1	26.6	4.5	22.2	5.9	26.1	5.3	37.3	9.7	21.6	3.9	0.9038(+)	0.8962(+)	0.7506(+)	0.7429(+)
Seasoned vegetables	8.7	4.1	6.7	0.9	9.6	1.6	9.3	1.6	8.4	1.3	10.0	1.6	8.8	1.5	8.2	1.6	8.8	2.2	6.0	1.4	0.9832(−)	0.9347(−)	0.4218(−)	0.4094(+)
Seasoned fresh vegetables	5.9	1.1	5.8	1.0	5.9	1.1	7.8	1.6	6.5	1.2	6.3	1.2	4.5	0.9	7.4	1.5	7.8	2.5	8.0	1.7	0.6071(−)	0.5215(−)	0.6189(+)	0.6574(+)
Kimchi	16.4	2.3	15.4	1.2	14.7	1.3	17.6	2.1	18.5	2.2	18.6	3.0	16.6	2.3	13.9	1.4	16.7	2.9	13.7	2.1	0.4537(−)	0.6448(−)	0.4009(−)	0.3618(+)
Pickled/preserved foods	3.1	1.5	3.0	0.9	2.2	0.5	1.6	0.4	1.6	0.5	1.6	0.5	0.9	0.3	1.0	0.4	1.8	0.6	0.3	0.1	0.0703(−)	0.0788(−)	0.0343(−)	0.0356(−)
Seasoning	4.6	1.0	8.0	2.5	6.3	1.6	9.2	2.1	7.7	1.6	6.0	1.6	6.4	1.9	6.4	1.8	9.9	2.2	12.1	3.1	0.8949(−)	0.9568(+)	0.1021(+)	0.1255(+)
Dairy products & ice creams	243.1	21.8	234.7	13.2	193.7	11.4	200.7	12.9	213.3	14.0	208.8	13.6	233.9	15.7	179.3	11.9	199.8	15.5	200.9	19.6	0.1326(−)	0.1734(−)	0.0694(−)	0.0536(−)
Beverages	91.8	8.2	92.4	8.5	105.7	9.7	104.7	10.2	94.5	8.5	108.4	11.0	81.4	9.4	85.6	10.1	109.8	14.3	68.1	11.4	0.3119(−)	0.4802(−)	0.2567(−)	0.4004(−)
Fruits	139.5	12.4	142.7	12.8	186.7	15.9	169.1	17.5	177.9	13.1	147.9	11.7	134.1	14.9	150.1	15.7	138.8	13.0	128.1	14.7	0.5631(−)	0.4564(−)	0.0799(−)	0.0482(−)
Sweets	6.4	1.4	5.0	1.1	5.7	1.0	7.3	1.4	5.4	1.0	8.1	1.6	9.2	1.4	9.8	1.3	8.3	1.5	9.1	1.7	0.0028(+)	0.0023(+)	0.0017(+)	0.0009(+)
Grain & potatoes	26.1	4.6	45.4	5.9	44.7	6.0	38.1	6.2	42.8	5.0	48.3	6.7	34.7	7.0	26.0	3.9	34.9	6.3	72.3	10.8	0.9895(−)	0.8615(+)	0.0547(+)	0.0566(+)
Pulses, nuts, & seeds	1.5	0.4	1.7	0.6	5.3	1.8	1.2	0.6	1.2	0.6	2.1	0.6	2.1	1.4	0.6	0.2	0.6	0.3	2.1	1.4	0.1058(−)	0.0392(−)	0.0842(−)	0.0653(+)
Vegetable & seaweeds	2.7	0.9	3.1	0.8	2.7	0.5	5.4	2.3	4.2	1.1	2.4	0.6	4.5	1.3	5.1	1.2	4.6	1.2	2.5	0.9	0.1198(+)	0.1738(+)	0.2923(+)	0.4994(+)
Fishes & meats	7.0	1.6	6.6	1.4	10.3	2.6	4.9	1.2	7.7	1.5	4.7	1.2	5.9	1.8	11.6	2.4	5.9	1.4	7.4	2.5	0.6076(+)	0.5087(+)	0.7321(−)	0.7418(+)
Others	0.1	0.1	0.1	0.1	0.1	0.1	0.5	0.4	0.1	0.1	0.2	0.1	0.1	0.1	0.1	0.1	0.1	0.03	0.1	0.1	0.9709(−)	0.8386(+)	0.3401(−)	0.2372(−)

^(^^1)^ *p* for trend by Generalized Linear Model (GLM), ^(^^2)^ Adjusted for gender, age, mother’s age, mother’s education level and energy intake.

**Table 6:** Top 20 foods contributing to the total food intake of Korean preschoolers by years’ daily food intake by dish group.

Rank	2012 (n = 282)			2013 (n = 313)			2014 (n = 282)			2015 (n = 209)			2016 (n = 331)		
	Food (g/day)	Mean	% ^(1)^	Food (g/day)	Mean	%	Food (g/day)	Mean	%	Food (g/day)	Mean	%	Food (g/day)	Mean	%
1	Milk	197.33	21.25	Milk	199.33	19.44	Milk	163.53	15.70	Milk	163.69	16.44	Milk	161.44	15.68
2	White rice	107.01	11.53	White rice	100.19	9.78	White rice	104.60	10.04	White rice	114.69	11.52	White rice	102.03	9.91
3	Apple	25.62	2.75	Apple	41.29	4.02	Apple	64.31	6.17	Apple	60.11	6.04	Apple	48.20	4.68
4	Mandarin	25.01	2.69	Mandarin	32.76	3.20	Egg	26.74	2.57	Egg	29.98	3.01	Yoghurt, curd type	29.84	2.90
5	Egg	24.43	2.63	Egg	27.47	2.68	Yoghurt, curd type	25.58	2.45	Yoghurt, curd type	28.25	2.84	Watermelon	28.94	2.81
6	Yoghurt, curd type	19.03	2.05	Fruit beverage	24.83	2.43	Pork	19.33	1.85	Fruit beverage	28.16	2.83	Egg	28.28	2.75
7	Fruit beverage	18.30	1.97	Pork	23.00	2.25	Bread	18.95	1.82	Mandarin	26.94	2.71	Fruit beverage	22.57	2.19
8	Potato	15.80	1.70	Bread	18.45	1.80	Watermelon	18.18	1.74	Bread	19.06	1.92	Mandarin	21.44	2.08
9	Watermelon	15.39	1.65	Yogurt, liquid type	18.18	1.78	Potato	17.80	1.71	Kimchi ^(2)^	18.66	1.88	Bread	21.09	2.05
10	Pork	14.28	1.54	Yoghurt, curd type	17.59	1.72	Grape	17.42	1.67	Potato	15.82	1.59	Yogurt, liquid type	18.78	1.82
11	Strawberry	13.66	1.47	Cracker, biscuit, cookie	17.55	1.71	Tofu	16.85	1.62	Pork	14.57	1.46	Pork	18.39	1.79
12	Chicken	13.59	1.46	Banana	15.98	1.56	Fruit beverage	16.65	1.60	Banana	14.48	1.46	Potato	18.33	1.78
13	Soybean milk	13.21	1.42	Watermelon	15.29	1.49	Chicken	15.84	1.52	Onion	14.18	1.43	Kimchi ^(2)^	15.74	1.53
14	Bread	13.10	1.41	Kimchi ^(2)^	13.70	1.34	Mandarin	15.58	1.50	Beef	13.76	1.38	Banana	15.20	1.48
15	Sherbet	12.16	1.31	Ice cream	13.66	1.33	Pear	15.51	1.49	Pear	12.62	1.27	Rice cake	15.19	1.48
16	Kimchi ^(2)^	11.92	1.28	Potato	12.70	1.24	Orange	14.77	1.42	Chicken	12.21	1.23	Beef	15.17	1.47
17	Beef	11.84	1.27	Rice cake	12.67	1.24	Banana	14.27	1.37	Yogurt, liquid type	12.13	1.22	Oriental melon	14.35	1.39
18	Tomato	11.82	1.27	Chicken	12.57	1.23	Onion	14.07	1.35	Other soft drinks	12.08	1.21	Orange	13.50	1.31
19	Yogurt, liquid type	11.81	1.27	Sweet potato	12.44	1.22	Sweet potato	13.96	1.34	Watermelon	11.90	1.20	Strawberry	13.39	1.30
20	Grape	11.72	1.26	Onion	12.32	1.20	Anchovy broth	13.60	1.31	Cucumber	11.38	1.14	Ice cream	12.05	1.17
**Rank**	**2017 (n = 278)**			**2018 (n = 246)**			**2019 (n = 235)**			**2020 (n = 179)**			**2021 (n = 154)**		
	**Food (g/day)**	**Mean**	**% ^(1)^**	**Food (g/day)**	**Mean**	**%**	**Food (g/day)**	**Mean**	**%**	**Food (g/day)**	**Mean**	**%**	**Food (g/day)**	**Mean**	**%**
1	Milk	179.05	17.02	Milk	178.51	18.12	Milk	148.20	15.41	Milk	167.47	15.83	Milk	190.21	18.86
2	White rice	109.82	10.43	White rice	103.15	10.46	White rice	103.16	10.72	White rice	99.66	9.43	White rice	105.48	10.46
3	Fruit beverage	46.37	4.41	Fruit beverage	38.07	3.87	Apple	36.85	3.83	Fruit beverage	43.89	4.15	Fish broth	46.96	4.66
4	Egg	33.06	3.14	Apple	30.62	3.10	Fruit beverage	32.95	3.42	Egg	35.95	3.40	Apple	33.73	3.35
5	Apple	30.84	2.93	Fish broth	29.68	3.01	Egg	30.65	3.19	Pork	32.25	3.05	Egg	31.65	3.14
6	Pork	27.07	2.57	Yogurt	28.40	2.88	Pork	27.09	2.81	Apple	31.79	3.00	Fruit beverage	29.69	2.95
7	Fish broth	25.88	2.46	Mandarin	26.08	2.64	Fish broth	26.03	2.70	Beef	23.19	2.19	Pork	27.65	2.75
8	Yogurt	20.70	1.97	Egg	25.77	2.61	Mandarin	24.92	2.59	Chicken	22.49	2.13	Kelp broth	20.93	2.08
9	Mandarin	19.59	1.86	Kelp broth	21.89	2.22	Bread	19.01	1.97	Fish broth	21.75	2.06	Yogurt	20.58	2.04
10	Chicken	17.36	1.65	Pork	21.81	2.21	Chicken	18.21	1.89	Kelp broth	21.37	2.02	Tofu	17.59	1.75
11	Bread	16.84	1.60	Bread	19.81	2.01	Kelp broth	17.48	1.81	Other sweetened drinks	20.59	1.95	Watermelon	16.90	1.68
12	Strawberry	16.59	1.58	Yogurt drink	19.11	1.94	Yogurt	16.76	1.74	Watermelon	20.14	1.90	Beef	16.48	1.64
13	Kelp broth	16.52	1.57	Potato	17.16	1.74	Beef	16.31	1.69	Bread	18.88	1.78	Potato	16.26	1.61
14	Yogurt drink	15.87	1.51	Beef	17.00	1.72	Potato	14.47	1.50	Black tea	18.81	1.78	Chicken	15.09	1.50
15	Kimchi ^(2)^	15.55	1.48	Peach	14.59	1.48	Other sweetened drinks	13.46	1.40	Grape	16.59	1.57	Bread	14.62	1.45
16	Beef	15.35	1.46	Tofu	13.30	1.35	Banana	12.90	1.34	Potato	15.52	1.47	Pear	14.27	1.42
17	Rice cake	15.15	1.44	Onion	12.75	1.29	Kimchi ^(2)^	12.71	1.32	Yogurt	14.68	1.39	Other sweetened drinks	13.81	1.37
18	Pear	14.59	1.39	Sherbet	12.42	1.26	Onion	12.45	1.29	Onion	14.33	1.35	Mandarin	13.28	1.32
19	Other sweetened drinks	14.08	1.34	Kimchi ^(2)^	12.01	1.22	Peach	12.36	1.28	Cracker	12.59	1.19	Strawberry	13.23	1.31
20	Onion	13.22	1.26	Chicken	11.88	1.20	Strawberry	12.05	1.25	Yogurt drink	12.54	1.18	Yogurt drink	12.60	1.25

^(1)^ Weighted %, ^(2)^ Napa Cabbage Kimchi.

## Data Availability

All data were obtained from the Korea Disease Control and Prevention Agency and are available with the permission of the Korea Disease Control and Prevention Agency. The data in this study were from the Korea National Health and Nutrition Examination Survey: https://knhanes.kdca.go.kr (accessed on 9 December 2025).
